# A dangerous balancing act: On matters of science, a well‐meaning desire to present all views equally can be an Trojan horse for damaging falsehoods

**DOI:** 10.15252/embr.201948706

**Published:** 2019-07-09

**Authors:** David Robert Grimes

**Affiliations:** ^1^ University of Oxford Oxford UK; ^2^ Queen's University Belfast Belfast UK

## Abstract

Journalistic impartiality is a laudable aim, but overly rigid application of unbiased reporting may do more harm than good. The issue of false balance in science reporting has severe consequences for health and the environment.

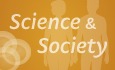

In an era of increasingly polarised discourse, journalistic impartiality is a virtue that media outlets should strive to achieve in order to provide readers and viewers with unbiased, neutral information. In a hyper‐partisan world, dedicated and balanced reporting is more vital than ever to cleave sound from fury, to help readers to make sense of conflicting narratives. But as laudable an aspiration as this is, overly rigid application can do more harm than good—and nowhere is this more obvious than on scientific topics.

Take, for example, climate change. The evidence for anthropogenic global warming is overwhelming. A wealth of data points to the same stark conclusion: our climate is rapidly changing, driven by human activity. This conclusion is not controversial among scientists; in fact, climate change denial is about as well‐supported as the obsolete concepts of spontaneous generation or phlogiston theory. Yet, denialist positions were afforded roughly equal media coverage as the scientific consensus. This dichotomy tremendously skewed public perception. While scientists are virtually in agreement on the reality and causes of climate change, up until recently approximately almost all articles in prestige American media gave equal coverage to climate change denialists as they did to scientific consensus 1.

## Skewing the balance

This is an example of false balance, which occurs when one tries to treat two opposing positions as equally valid when they are simply not. If one position is supported by an abundance of evidence while another is entirely bereft of it, it is profoundly misguided to afford equal air‐time and coverage to both positions. And yet, in attempting to maintain impartiality, this is precisely what many outlets end up doing. Even an institution as August as the BBC has fallen victim to this error. In 2011, they were harshly criticised by the BBC Trust for “undue attention to marginal opinion” on climate change, due to “over‐rigid application of editorial guidelines on impartiality”. The net result was climate change deniers being afforded far too much air‐time. A 2014 follow‐up found that this key conclusion “still resonates today” 2.

If one position is supported by an abundance of evidence whilst another is entirely bereft of it, it is profoundly misguided to afford equal air‐time and coverage to both positions.

False balance is insidious, giving dubious positions an illusion of respectability. While well‐intended, it is all too frequently a Trojan horse that allows the most odious of fictions to gain a foothold. False balance creates a perception in the public mind that an issue is scientifically contentious, when it is not. This ultimately means that even urgent issues such as climate change can be dismissed as a mere difference of scientific opinion. This confounds not only public perception of science, but creates an aura of doubt, which can be abused to create a toxic inertia, beguiling us to sleep‐walk into disaster or placidly accept situations we should vocally protest.

This has long been exploited by the duplicitous. Decades ago, tobacco companies realised confounding public understanding was their strongest defence against the inescapable evidence that their product was highly toxic. A now infamous internal memo from 1969 makes for ominous reading: “Doubt is our product since it is the best means of competing with the ‘body of fact’ that exists in the mind of the general public. It is also the means of establishing a controversy”. Tobacco executives and the public relation firms they engaged stoked a manufactured controversy, which succeeded for decades to lure the general public into a perception that the health risks of smoking were unclear, when the scientific evidence was unambiguous 3.

That revealing memo remains a blueprint for those eager to negate overwhelming scientific consensus. Parallels with contemporary efforts by the fossil fuel lobby to downplay climate change are hard to overstate—they essentially read from the same script. These devious tactics rely on exploiting the journalistic desire for balance. And to be fair to journalists, striving to eliminate bias is admirable. In matters of opinion and politics, treating opposing views as equally worthy of merit is generally a sensible approach. But this policy breaks down utterly for scientific issues, where positions and consensus are crafted based on the preponderance of evidence. If one position is buttressed by an overwhelming weight of evidence while another is bereft of empirical support, it is profoundly wrong‐headed to treat them as equal. And yet, this is precisely what occurs all too often in the coverage of scientific issues.

## The ideological Trojan Horse

The tobacco and fossil fuel industries have obvious financial incentives to muddy the waters. But false balance is frequently the unwitting ally of ideological positions bereft of evidence, occurring in arenas with no obvious financial interests. Evolutionary theory is the bedrock of modern biology. To biblical creationists however, it is seen as borderline blasphemous as it contradicts biblical accounts of the dawn of mankind. In 1999, religious conservatives began to promote “Intelligent design” (ID) as an alternative to evolution by natural selection. It was nothing more than a rebranding of creationism with the pretence of science. Even so, its promoters argued that as evolution was merely a theory, then their theory was equally valid, and should be taught alongside evolution.

Anti‐vaccine activists have proven extraordinarily adept at exploiting false balance to evangelise their discredited views.

This conflation was extremely disingenuous, pivoting on the ambiguity of the word theory. In colloquial context, theory is akin to “idea” or “supposition”. A scientific theory, however, is not mere conjecture but something supported by multiple strands of evidence and solid data. Evolution is “just” a theory in the same way germ theory or the theory of relativity are “just” theories. ID had no such claim to the word, yet the basis of their “wedge strategy” was to exploit false equivalence. Despite the “Teach the Controversy” campaign being slammed by the American Association for the Advancement of Science, the Dover Area School District taught ID alongside evolution, until a legal challenge in 2005 ruled that teaching ID as equivalent with scientific theory was wholly unjustified 4.

The posturing of the intelligent design lobby might be laughable, but false balance can wrack terrible consequences—noticeably when human health is involved. Vaccination is such a flashpoint issue, and after clean water, nothing has saved more lives. It has hugely reduced the burden of infectious disease, banishing once ubiquitous scourges like small‐pox to the confines of history. And yet, immunisation has faced militant opposition right from the beginning. Resistance to public health efforts and scientific progress is largely ideological in nature and resistant to correction. Efforts to change the minds of dedicated anti‐vaccine activists frequently backfire, entrenching them further in their ill‐founded views. As the World Health Organisation wearily notes, “How one addresses the anti‐vaccine movement has been a problem since the time of Jenner. The best way in the long term is to refute wrong allegations at the earliest opportunity by providing scientifically valid data. This is easier said than done, because the adversary in this game plays according to rules that are not generally those of science 5”.

## A licence to scaremonger

Anti‐vaccine activists have proven extraordinarily adept at exploiting false balance to evangelise their discredited views. The measles–mumps–rubella (MMR) vaccine controversy is an infamous illustration. In 1998, English gastroenterologist Andrew Wakefield held a press conference about a paper he had published in the Lancet, speculating on a link between autism and the MMR vaccine. His evidence, however, was extraordinarily weak. Mainstream science and health writers noted such an explosive charge was poorly justified, and the story initially received scant attention. Anti‐vaccine activists instead pitched it to non‐specialist writers as a human interest story, imploring journalists without any scientific training to pontificate on the ostensible link between autism and the vaccine, and to report “both sides”.

This proved a devastatingly effective strategy. By the year 2000, a whole 10% of all science stories in the UK were related to MMR, over 80% of them authored by non‐science journalists. In the words of physician and writer Ben Goldacre, “Suddenly we were getting comment and advice on complex matters of immunology and epidemiology from people who would more usually have been telling us about a funny thing that happened with the au pair on the way to a dinner party”. There was virtually no evidence that the vaccine was harmful, and an abundance of data showing it to be safe and effective. While public health bodies and scientists desperately tried to convey this reality, editors and writers lacked the requisite scientific background to weigh up the strength of evidence for these opposing claims. Presented with two wildly different narratives, they fell back upon the default assumption that mutually opposed views must have equal merit, warranting equal coverage. The resultant framing of the MMR vaccine as controversial was hugely damaging to public confidence, and uptake fell markedly.

A study or claim in isolation cannot be fully understood without the requisite context and background, and yet too often this is completely absent in coverage.

Consequences were devastating: vaccination rates across Western Europe fell well below the 94% immunisation levels required to stem the onslaught of measles, a disease so virulent each single case tends to produce 12–18 secondary infections. A spate of cases ensued across the UK and Ireland, resulting in the deaths of innocent children. Around the same time, investigative journalist Brian Deer turned a more sceptical eye to Wakefield's claims than the hagiography of many of his peers. His investigations ultimately exposed Wakefield's autism–MMR link as fraudulent and unveiled financial and ethical conflicts of interest. With his work shown to be falsified, the paper was retracted, and Wakefield struck off for unethical behaviour. The damage, alas, was sadly done, with long‐term consequence. Measles, once on the verge of eradication, has again taken a foothold around the world with record number of cases in both Europe and America this year, prompting the WHO to declare vaccine hesitancy a top 10 threat to public health.

## Lessons unlearnt

Wakefield may have the lion's share of blood on his hands for the MMR vaccine debacle, but false balance allowed him to perpetuate such fatal mendacity. We are still trying to reckon with the fall‐out of that disaster, while the next one is already affecting the human papillomavirus (HPV) vaccine. The HPVs include more than 170 virus strains, some of which can cause genital cancers. Virtually, all cervical cancers are HPV‐mediated, and as the vaccine protects against the most dangerous strains, it has the potential to end the misery of cervical cancer and its odious siblings. This is not hyperbole—Australia, an early adopter, is on track to eliminate cervical cancer by 2028, and precancerous infections have fallen the world over in countries with high uptake of the vaccine 6.

But worryingly, public trust in the HPV vaccine has recently been undermined in several countries, and false balance reporting has contributed to it. In Japan, media reports of anti‐vaccine claims led to mass panic in 2013. Even though subsequent investigation showed the vaccine to be safe and effective, reporting of purported dangers based on hearsay diminished uptake from 70 to 1% in 1 year. In 2014, Denmark was hit by similar panic, with anti‐vaccine claims given an equal airing in national media, leading to uptake falling from 79% to < 17% in a year. By 2015, HPV vaccine panic came to Ireland when an anti‐vaccine group successfully courted media attention. The ensuing attention made the vaccine appear controversial in the public eye, and vaccine uptake fell from highs of 87% to around 50% within a year.

When scientists abuse their position to push anti‐scientific nonsense, it adds hugely to public confusion. This is perhaps something that the scientific community should take a more active role in addressing.

This trend has finally begun to reverse in Ireland at least, after sustained efforts by scientists, physicians, patient advocates and public health bodies 7. But the fact that long‐discredited anti‐vaccine claims still cause damage should not surprise us. There is ample evidence that anti‐vaccine campaigners are especially adept at spreading misinformation online, entirely unconcerned with the veracity of what they propagate. This is a small but vocal minority—most parents who are vaccine‐hesitant are not anti‐vaccine zealots, but simply concerned, unsure of how to parse the cacophony of claims to which they are subjected. It is completely understandable that parents can become apprehensive, particularly when media reporting all too often presents the topic as contentious.

## The road to hell is paved with good intentions

So why does this failing occur so persistently? The crux of the problem stems from a fundamental confusion over what science is and what it is not. Quality scientific reporting requires an understanding of the scientific method and an implicit grasp of a subject. A study or claim in isolation cannot be fully understood without the requisite context and background, and yet too often, this is completely absent in coverage. The dark irony of false balance is that it is a bias, which usually arises from a concerted attempt to avoid bias. Yet when it comes to issues of science, striving for balance is mistaken when accuracy is a much more appropriate aim. To quote Boyce Rensberger, “balanced coverage of science does not mean giving equal weight to both sides of an argument. It means apportioning weight according to the balance of evidence”.

Science of course is not an argument from authority; the hypotheses of even Nobel laureates can be utterly debunked by the experiments of the humblest student. Nor is it a popularity contest; scientific consensus is derived based on the strength of evidence for a given position. Scientists, when they are practising science, only speak with any authority when they are reflecting best evidence. If they instead advocate a position unsupported by the evidence, their qualifications are utterly irrelevant. Sadly, there are plenty of scientists and physicians who push discredited views, abusing their credentials to bamboozle; Wakefield's MMR manipulations; Linus Pauling's promotion of vitamin C as a universal panacea; and Peter Duesberg's AIDS denialism. When scientists abuse their position to push anti‐scientific nonsense, it adds hugely to public confusion. This is perhaps something that the scientific community should take a more active role in addressing.

Writing on false balance for the *Columbia Journalism Review* in 2004, Chris Mooney elucidated how the ideal of impartiality can give odious falsehoods a veneer of legitimacy they simply do not deserve: “As a general rule, journalists should treat fringe scientific claims with considerable skepticism, and find out what major peer‐reviewed papers or assessments have to say about them. Moreover, they should adhere to the principle that the more outlandish or dramatic the claim, the more skepticism it warrants”. Of course, it takes expertise to gauge the merit of scientific claim, and journalists are under tremendous pressure to produce engaging stories in an era that values velocity over veracity. It is not realistic to expect media outlets to have the same level of understanding of a field that a researcher does and differentiating between valid science and pseudoscientific nonsense can be remarkably difficult. To compound matters, misinformation and dedicated lobbying make it exponentially harder for media outlets to discern the truth on difficult topics.

## The age of disinformation

This vacuum of knowledge is all too easy for vested interests to hijack, engineering a platform for unsubstantiated views. Moreover, since Mooney's observations 15 years ago, our new channels have drastically changed. The Internet has become our primary source for information, frequently filtered through the distorting prism of social media. The impact has been stark: the media has become far more fragmented, and messages which promote outrage or emotional reaction tend to be much more widely shared, regardless of their veracity 8. The traditional triumvirate of newspapers, television and radio has been supplanted largely by Internet‐based publications. This has positive aspects and has certainly diversified sources of information. But recent investigations into Russian disinformation campaigns have illustrated how this greater ecosystem of information channels allows one to conjure misinformation wholesale, readily disseminating it without the typical machinery of the press. Consequently, one can bypass traditional media channels completely, perpetuating any falsehood desired.

The media has become far more fragmented, and messages which promote outrage or emotional reaction tend to be much more widely shared, regardless of their veracity.

Dubious sources are alarmingly common, often outnumbering reputable accounts 9. Nor are we especially adept at identifying questionable sources; one Stanford study investigating this labelled their findings “bleak” and “a threat to democracy” 10. With fringe sources that deliberately blur the line between information and propaganda, false balance is no longer the issue, as these sources eschew any pretence of impartiality. But the damage done can be further compounded by reputable outlets engaging in false balance; this often occurs when journalists deem a claim worthy of coverage due solely to a volume of claims about it, rather than the quality of those claims. In doing so, they can lend legitimacy to positions that do not warrant any oxygen, cementing skewed perceptions in the public mind. This “feedback loop” form of false balance has certainly been at the root of a number of vaccine panics in recent years and has equally driven fears over genetically modified food and 5G technology.

## The crucial role of informed reporting

But despite these failings, media outlets have a more vital role to play than ever before. Reputable outlets have a commitment to fact‐checking and impartial analysis. Informed reporting is an invaluable shield against the onslaught of falsehoods. By maintaining a standard for fact‐checking and quality control, media outlets can be a bulwark against the undue sway of increasingly partisan sources. Rather than lament failings, it might be more productive for scientists to engage more with the media so that false balance might be circumvented. A promising way to reduce poor reportage on scientific issues is to put scientific experts in direct contact with those covering the story. This is an approach taken by organisations such as the Science Media Centre and Sense About Science. For journalists, an index of knowledgeable experts they can approach to gauge a claim and put it in context is invaluable, and this is beneficial on a societal level. Aside from more informed reporting, I have found that this can kill scaremongering and fictitious claims before they become a story.

Increased engagement with media outlets is mutually beneficial, improving both understanding and appreciation of science and medicine.

Knowing when to engage, however, is a more nuanced question. There is still a fixation with sensationalism, and a fallacious idea that debate rather than discussion is the arbiter of truth. This is abject nonsense—too frequently, debate rewards those with the most devious rhetorical skills and the greatest propensity to lie. I have lost count of the times I have been contacted by an outlet, wondering if I would debate someone who denies the reality of climate change, or an ardent anti‐vaccine activist. These days, I steadfastly refuse to do so, explaining that this would be textbook false balance, and would lend a sheen of respectability to odious views. For their part, media outlets need to appreciate that in matters of science, an adversarial talking heads’ format frequently produces much more heat than light, leaving audiences unsure and divided. Public understanding is ultimately much better served by discussion and explanation rather than oratorical theatrics.

Crucially, it is entirely possible for media outlets to cover contentious topics in an informative and responsible manner, with requisite training. The BBC has admirably instituted a policy of training its reporters to avoid false balance on complex and contentious issues in science, noting that “… science coverage does not simply lie in reflecting a wide range of views but depends on the varying degree of prominence (due weight) such views should be given”. The problem of false balance is not likely to dissipate anytime soon in our hyper‐partisan and polarised media landscape. Effectively addressing this certainly requires a concerted change in media behaviour. Informed discussion is far more valuable than mindless exercises in false equivalency.

Responsibility for improving societal understanding is not the media's alone. If we are to stem the tide of misinformation, it is imperative that scientists themselves become more enmeshed in the communication of science. Increased engagement with media outlets is mutually beneficial, improving both understanding and appreciation of science and medicine. We cannot forget that we live in an era where falsehoods can perpetuate rapidly; once a myth has taken hold, countering it becomes nigh on impossible. A more proactive stance from the scientific community is vital to stop fabrications taking root in the first instance. To shun this responsibly is to leave society more divided and less informed, to our collective detriment.

## Conflict of interest

The author declares that he has no conflict of interest.
